# The relation of dental students’ learning styles to their satisfaction with traditional and inverted classroom models

**DOI:** 10.1186/s12909-019-1749-x

**Published:** 2019-08-22

**Authors:** Rong Wang, Chuanyong Liu

**Affiliations:** 1 Department of Physiology and Pathophysiology, Shandong University, Cheeloo College of Medicine, Jinan, 250012 China; 2 Key Lab of Mental Disease, Shandong Province, Jinan, 250012 China

**Keywords:** Learning style, Student satisfaction, Instruction approach, Inverted classroom model

## Abstract

**Background:**

The authors’ medical school has adopted an inverted classroom model (ICM) for physiology classes. This study aimed to determine students’ learning styles and investigate the relationship between learning style and satisfaction with different instruction approaches and components of the ICM.

**Methods:**

One hundred and twenty-one second-year dental students participated in this study, which had a 77.6% participation rate. The Kolb Learning Style Inventory, a sociodemographic questionnaire, and a satisfaction survey were administered after course completion.

**Results:**

In both the traditional and ICM classes, most of the participants were convergers (56.9 and 54%) and assimilators (20.7 and 25.4%), and the rest of the participants were accommodators (15.5 and 12.7%) and divergers (6.9 and 8%). Learning style did not influence participants’ satisfaction and did not predict their satisfaction with the traditional and ICM approaches. The satisfaction scores for the four components of the ICM were not significantly different by learning style. The mean satisfaction scores of the ICM approach were higher than those of the traditional approach in all learning style groups. All of the participants in the ICM class were more satisfied with the online and teacher-student interaction components than the student group discussion and presentation components.

**Conclusions:**

Learning style may not be a potential contributing factor for optimizing the implementation of the ICM. Instead of focusing on learning styles, further research must investigate how to design more efficient online courses, determine appropriate levels of learning materials, provide more online instructional interaction, and help students overcome their feelings of fear.

**Electronic supplementary material:**

The online version of this article (10.1186/s12909-019-1749-x) contains supplementary material, which is available to authorized users.

## Background

Successful medical students are expected to have the ability to organize and manage their learning. Self-learning skills are particularly crucial to achieving effective lifelong learning in the medical field, where scientific knowledge is continuously producing and changing [1]. Therefore, medical schools have started to adopt new teaching methods, such as the inverted classroom model (ICM), to train medical students to be effective self-learners. Some reports have indicated that student engagement under the ICM results in improvement in self-learning skills [2, 3] and greater facilitation of student-centred learning [4–6]. However, the question remains as to how the design and implementation of the ICM could be maximized for efficiency and satisfaction outcomes. Since students are the centre of medical education, information about the individual learning characteristics of students, such as their learning styles, might help answer such questions. Learning styles theory indicates that if a teaching method is matched to the learning styles of the learners, educational outcomes can be improved. However, there are continuing disputes over the use of the learning styles theory because of the lack of evidence to support it [7–9]. Interestingly, despite this lack of evidence, there is a widespread belief about the value of the use of learning styles in education [9]. Some studies have demonstrated the popularity of the use of learning styles among school teachers, including higher education faculty [10–12]. Similarly, in our teaching practice in China, most faculty believe that teaching according to a student’s learning style can enhance learning, and they have tried to make recommendations for curriculum reform, for instance, about how to improve the quality of the ICM based on learning styles theory. Therefore, as a first step towards exploring the possibility of solving this problem, we aim to validate the ability of learning style instruments to improve the quality of the ICM.

More than 70 different learning styles schemes have been formulated [13]. Kolb’s learning styles constitute one of the best-known and widely used learning style theories. David Kolb’s model is the basis for the Learning Style Inventory, an assessment tool to categorize learners as to “how one acquires knowledge” [14]. According to Kolb’s model, individuals may prefer one of four styles — Accommodating, Converging, Diverging, or Assimilating (Additional file 1: Figure S1)— depending on their approach to learning [14]. Kolb suggested that learning is a four-stage circle from experience to observation to conceptualization to experimentation and back to experience (Additional file 1: Figure S1). Despite a lack of evidence to support the use of learning styles, some educators have supported the argument that learning about students’ individual learning styles is essential for faculty to design and implement effective teaching [11, 15]. In addition, some medical faculty in higher education have reported that the learning styles from Kolb’s LSI were related to satisfaction or preferences for certain instruction approaches [16, 17].

Many studies have reported the positive impact of the ICM on students’ performance and perceptions [18–21]. Regarding problem-based learning (PBL) classes, there have been studies on the relationship of medical school students’ learning styles to their satisfaction [16, 17]. However, in the context of ICM classes, no study has assessed dental students’ satisfaction in terms of their learning styles. To explore a possible strategy for more effective implementation of the ICM, this study aimed to identify the learning styles of dental students in traditional and ICM classes at Cheeloo College of Medicine, Shandong University, and investigate the relationship between students’ learning styles and their satisfaction with different instruction methods and the different components of the ICM.

## Methods

### Participants and ethics

Medical physiology is taught to 2nd-year dental students at Cheeloo College of Medicine, Shandong University. This study compared a traditional lecture course taught in fall 2015 and an ICM-based course taught in fall 2018. All students from both classes (n = 156) entered the study (without any sampling). Of both groups, 121 students completed the questionnaire and inventories appropriately, for a participation rate of 77.6%. No pedagogic changes in the teaching syllabus, textbook, or learning materials, such as problem sets, were made to the teaching contents of the course. This study, with use of the student survey, was identified as exempt from supervision by the Ethics Committee of Shandong University, Cheeloo College of Medicine. Students were informed about the study and signed consent forms.

### Study design

We used a comparative design. The traditional and ICM classes were regarded as two different instruction approaches. The two different teaching models are compared in Table 1. The online component of the ICM, the medical physiology massive open online course (MOOC), was designed by the Department of Physiology of Shandong University on the Icourse platform (http://www.icourse163.org/course/sdu-437005#/info). In this study, satisfaction with different instruction approaches or components of the ICM were the dependent variables, and learning styles, educational background of the parents, and gender were independent variables.

**Table 1:** Comparison of the traditional and ICM teaching models. Table 1–1 is shown in. Additional file 2

Implementation	Traditional Class	ICM Class
Teaching pattern	Four sections for 13 weeks: 1. Online course 2. Group discussion (F2F time: 10 mins per week) 3. Presentation (F2F time: 50 mins per week) 4.Teacher-student interaction (F2F time: 40 mins per week)	One section for 13 weeks: 200 min of instruction in a F2F time lecture format per week.
Learning contents	Textbook: twelve thematic chapters structured on the basis of organ system-related themes.	Online course: twelve thematic blocks structured on the basis of organ system-related themes, each with multiple sub-block including one course outline, one to three less than 15-min in length micro-lesson videos, which cover one or two main points of one sub-block (an example of one sub-block showed in Table 1–1).
Before F2F time	Preview the study materials by students (Textbooks, etc.).	Self-study online course by students (Access the online part on personal computer or smartphone.): 1. Watch micro-lesson videos selected from MOOC. 2. Read materials, online homework, one online quiz (Multiple-choice questions and Long and short-answer questions). And participate in a discussion board.
In F2F time	Teacher-centered teaching by using the multimedia teaching, 200 mins per week.	1. Group discussion (10 mins per week) 2. Presentation (50 mins per week) 3.Teacher-student interaction through an audience response system software (40 mins per week).
After F2F time	Homework and feedback from instructors	Review the learning contents and acquire additional resources from discussion board.
Grade	Final examination (80%), usual performance (20%).	Final examination (70%), online credit (30%).

### Data collection

The surveys were conducted with both classes of dental students using Kolb’s Learning Style Inventory (LSI), the satisfaction questionnaire, and the simple sociodemographic questionnaire at the end of the course.

#### Kolb’s learning style inventory (LSI)

The LSI, developed by Kolb, is a widely distributed tools to assess individual learning styles [14]. According to the results of studies on the reliability and validity of Kolb’s Learning Style Inventory (LSI) scores that have been conducted in several countries, including China, a Cronbach’s alpha value of 0.7 was used for the Kolb’s LSI used in our study.

Kolb’s LSI was used to collect data about the students’ learning styles. The LSI contains four primary subscales that assess concrete experience (CE), reflective observation (RO), abstract conceptualization (AC), and active experimentation (AE) (Additional file 1: Figure S1). The AE and RO constructs are from left to right on the horizontal axis, and the CE and AC constructs are from top to bottom on the vertical axis. In the inventory, respondents are asked to rank four phrases that correspond to the four learning modes. A score of four is given to the phrase that is considered to be the most characteristic of a respondent’s learning style, whereas a score of one is given to the phrase that is considered to be the least characteristic of his/her learning style. The subscale scores are combined to assess an individual’s preference for abstractness over concreteness (AC–CE) and action over reflection (AE–RO). Then, the preference scores are plotted on the horizontal and vertical axes and fall within one of the four quadrants, with each quadrant representing one learning style (Additional file 1: Figure S1).

Students with the four learning styles can be described as follows. First, accommodators mainly do, watch, and listen when they learn. Second, divergers are dominant in concrete experience (CE) and reflective observation (RO). They mainly observe and are open to experiences when they learn. Third, convergers like ideas, theories, and doing when they learn. Fourth, assimilators mainly think and watch and are interested in abstract concepts; they use logic to define a problem.

#### Satisfaction questionnaire

The satisfaction questionnaire was designed by the authors to identify the satisfaction levels of participants with different approaches and different activities involved in the ICM the 10 items of the questionnaire were developed based on the CEQ (Course Experience Questionnaire) used in Australia [22] and referenced a previously published empirical research paper [16]. For each of the ten statements on the questionnaire, students were asked to check the number (on a Likert scale between 5 = strongly agree and 1 = strongly disagree) that best reflected their rating of the traditional approach, the ICM approach and different activities involved in the ICM. Of the ten items, six statements were about how students felt about the two different instruction approaches, including their psychological comfort, and how the approaches contributed to the students’ learning and their future professional lives. The mean satisfaction score of each student was the student’s total score from the six statements divided by 6. Overall satisfaction was estimated with the mean score of the six statements in the satisfaction questionnaire. Then, to measure the level of satisfaction with each approach, students whose mean score was above 3.0 were considered satisfied with the approach. The other four statements were about how the different components of the ICM contributed to learning (Table 4).

### Data analysis

Statistical analyses were performed by using GraphPad Prism 6 software (Prism 6.0, GraphPad Software, San Diego, CA, USA) and SPSS 23.0 (IBM Corp., Armonk, NY, USA). To identify differences in satisfaction scores among students with the four learning styles and with different instruction approaches and components of the ICM, independent-samples t-tests and one-way ANOVA were used as appropriate. The basic principle for using nonparametric tests (Mann-Whitney test and Kruskal-Wallis test) in some comparisons was an inadequate number (< 30) of participants. The general linear model-based univariate ANOVA technique was used to assess the effects of students’ learning styles on their satisfaction with the different instruction approaches and their satisfaction with the four components of the ICM. To evaluate the predictive effect of the four learning styles of students on satisfaction with different instruction approaches, we used binary linear logistic regression analysis. A probability value of P < 0.05 was considered to be significant.

## Results

### Learning style inventory

A total of 58 (response rate 76%) and 63 (response rate 79%) participants in the traditional and ICM classes, respectively, completed this inventory. The average ages of the student populations were 19.1 ± 0.7 yr. and 19.1 ± 0.5 yr., respectively. The characteristics of the participants in the two classes are shown in Table 2. Of the 58 students in the traditional class, the largest group by learning style was the converger group, representing 56.9% (n = 33) of the class. The remaining students in the assimilator, accommodator and diverger groups represented 20.7% (*n* = 12), 15.5% (n = 9) and 6.9% (n = 4) of the class, respectively. Of the 63 students in the ICM class, the largest group by learning style was the converger group, making up 54% (*n* = 34) of the class. The rest of the students in the assimilator, accommodator and diverger groups represented 25.4% (*n* = 16), 12.7% (n = 8) and 8% (n = 5) of the class, respectively (Additional file 1: Figure S1).

**Table 2:** Characteristics of the participants

	Gender		Father′s educational level		Mother′s educational level	
	Female	Male	High school or lower	University or higher	High school or lower	University or higher
Traditional approach				*n* (percentage)		
Converger	18 (51.4)	15 (65.2)	15 (55.6)	18 (58)	17 (48.6)	16 (69.6)
Assimilator	7 (20)	5 (21.7)	5 (18.5)	7 (22.6)	9 (25.7)	3 (13)
Accommodator	8 (22.9)	1 (4.3)	5 (18.5)	4 (12.9)	6 (17.1)	3 (13)
Diverger	2 (5.7)	2 (8.7)	2 (7.4)	2 (6.5)	3 (8.6)	1 (4.3)
Total	35 (100)	23 (100)	27 (100)	31 (100)	35 (100)	23(100)
ICM approach				*n* (percentage)		
Converger	22 (61)	12 (44.4)	23 (60.5)	11 (44)	21 (53.8)	13 (54.2)
Assimilator	6 (16.7)	10 (37)	8 (21.1)	8 (32)	10 (25.6)	6 (25)
Accommodator	5 (13.9)	3 (11.1)	4 (10.5)	4 (16)	6 (15.4)	2 (8.3)
Diverger	3 (8.3)	2 (7.4)	3 (7.9)	2 (8)	2 (5.1)	3 (12.5)
Total	36 (100)	27 (100)	38 (100)	25 (100)	39 (100)	24 (100)

The percentage values represent the total number of students (n) per learning style group in the traditional and ICM classes

### Student survey results

**Fig. 1:**
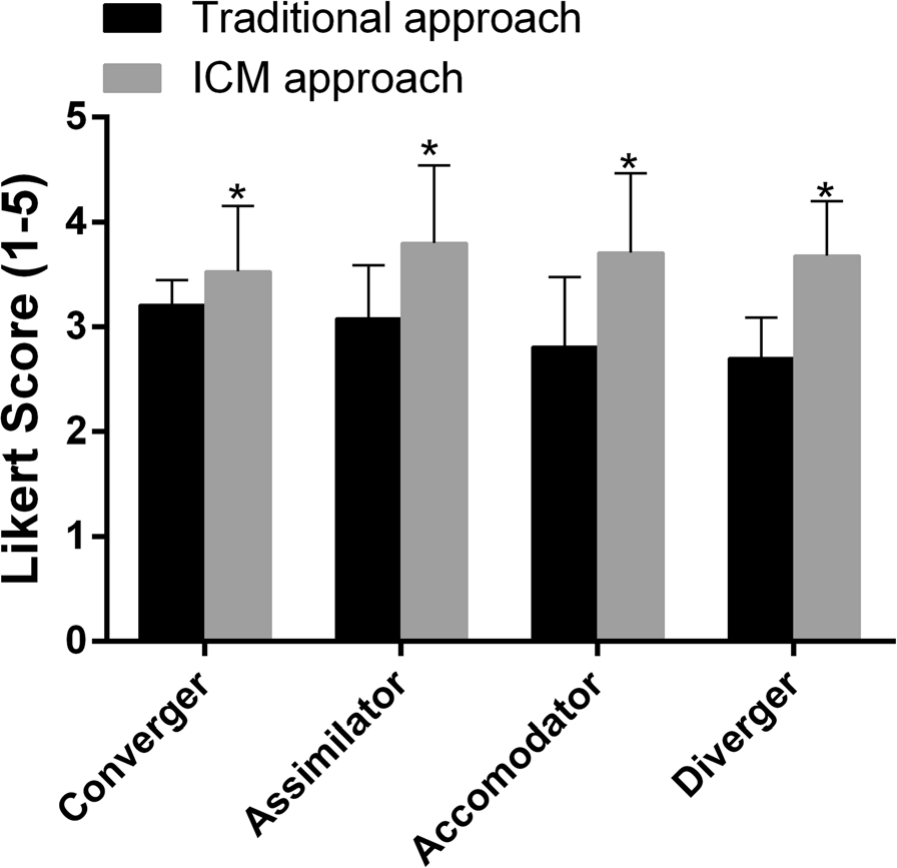
Students’ satisfaction with the instruction approach (traditional and ICM approaches) by learning style. The satisfaction score for each approach is the mean score of the six questionnaire statements listed in Table 3. The responses to each of six statements were provided using a Likert scale ranging from 5 to 1 (i.e., strongly agree, agree, unable to comment, disagree, and strongly disagree). The values are presented as the means ± SD. *P < 0.05, analysis by independent-samples t-test

For the 6 statements in the satisfaction questionnaire (a Cronbach’s α-value of 0.85 for the questionnaire), the mean scores for the ICM approach were higher than those for the traditional approach in all learning style groups (Fig. 1). Of the six statements, five had higher mean scores for the ICM approach than for the traditional approach; only the statement “I spend less time on learning this course” had a higher score for the traditional approach, which was the highest satisfaction score (3.5) for the traditional approach. However, the highest satisfaction score (3.9) was given by participants in the ICM class in response to the statement “The course format is helping me to better prepare for my future professional life” (Table 3).

**Table 3:** Students’ satisfaction with the instruction approach (traditional and ICM approaches)

Statements	Likert score	
	Traditional approach	ICM approach
1. The instruction approach currently used contributes to my learning course content	3.1 ± 0.5	3.7 ± 1*
2. I feel relax and comfort in this approach	3.1 ± 0.5	3.7 ± 0.9*
3. The course format is helping me to prepare my future professional life better	2.9 ± 0.5	3.9 ± 0.7*
4. The course format is helping me to prepare my exam better and receive higher score	2.9 ± 0.5	3.3 ± 0.6*
5. I spend less time on learning this course	3.5 ± 0.6*	3.2 ± 0.8
6. The course format is helping me to improve my communication skills	3.1 ± 0.6	3.5 ± 0.9*

The responses to each of six statements were assessed using a Likert scale ranging from 5 to 1 (i.e., strongly agree, agree, unable to comment, disagree, and strongly disagree). The values are presented as the means ± SD. *P < 0.05, analysis by independent-samples t-test

The results from the tests of between-subjects effects (Additional file 3: Table S2) indicated that the mean satisfaction scores were significantly different for the two different instruction approaches [F _(1,113)_ statistic (=30.98), P < 0.01]. The mean satisfaction scores were not different by learning style [P = 0.58]. Regarding the interaction effect of learning style and instruction approach, the impact of different learning styles on satisfaction was the same for students in the traditional class as it was for students in the ICM class [P = 0.08].

Binary logistic regression analysis was used to explain the relationship between satisfaction and learning style in traditional and ICM approaches. Learning styles were not predicting factors of satisfaction for the traditional [P = 0.10] or ICM approach [P = 0.90] (Additional file 4: Table S3).

According to the participants’ responses to the 4 statements on the satisfaction questionnaire (a Cronbach’s α-value of 0.84 for the questionnaire) about the different components of the ICM, in all learning style groups, the participants agreed more with statements 1 and 4 (“The online portion of the ICM class contributes to my learning the course content” and “The teacher-student interaction in the class meetings contributes to my learning the course content”) than with statements 2 and 3 (“Student discussion contributes to my learning the course content” and “Student presentations contribute to my learning the course content”) (Table 4). The results from the tests of between-subjects effects (Additional file 5: Table S4) indicated that the four dependent variables, i.e., the satisfaction scores for the four components of the ICM, did not differ significantly by learning style [P = 0.25, P = 0.24, P = 0.06 and P = 0.07].

**Table 4:** Satisfaction of the participants with 4 components of the ICM class (*n* = 63)

Satisfaction of participants with different activities associated with ICM class in ICM approach				
	Likert score for Statements			
	1 The online part of ICM class contributes to my learning course content	2 Students discussion section contributes to my learning course content	3 Students presentation section contributes to my learning course content	4 Teacher-student interaction section on the class meeting contributes to my learning course content
Converger	4.6 ± 0.6	3.6 ± 0.9^*^	3.5 ± 0.9^*^	4.2 ± 0.8
Assimilator	4.5 ± 0.5	3.6 ± 0.9^#^	3.6 ± 0.8^#^	4.1 ± 0.6
Accommodator	4.4 ± 0.7	3.3 ± 0.9^#^	2.8 ± 0.9^#^	3.8 ± 0.7
Diverger	4.0 ± 0.7	2.8 ± 0.8^#^	2.4 ± 0.5^#^	3.4 ± 0.5

The values are presented as the means ± SD. *P < 0.05, analysis by one-way ANOVA, ^#^ *P* < 0.05, analysis by Kruskal-Wallis test

## Discussion

By using Kolb’s LSI, our findings showed that, both in the traditional and ICM classes, most of the participants were convergers (56.9 and 54%) and assimilators (20.7 and 25.4%) who preferred to learn by forming abstractions, which is consistent with the results of some studies conducted among medical students and what would be expected in the area of natural sciences [23–25]. In both groups, although the four different learning styles were very unequally distributed, more than half of the students could be assigned to the converger type, and the distribution of participants’ preference scores along the two dimensions of Kolb’s LSI was scattered and broad (Additional file 1: Figure S1); hence, these participants could be further analysed to predict their satisfaction with different teaching approaches.

The results of our study showed that learning styles did not influence participants’ satisfaction with the instruction approach in the traditional or ICM classes. Learning styles may not predict satisfaction with traditional and ICM approaches. Our results do not support the use of learning styles to inform instruction. Instruction based on learning style has become prevalent in public education for the last two decades [7]. Based on a large amount of literature in the form of books, training materials, practitioner guides, and theoretical articles, many educators believe the learning styles hypothesis, that is, that matching the learning style to the instructional mode produces better academic and perceived performance (student satisfaction can be defined as a function of perceived performance regarding the service quality of the university [26, 27].). Some empirical studies have provided positive results to support this hypothesis [16, 17, 28–31].

On the other hand, many studies have refuted the hypothesis, suggesting weak associations between learning styles and learning outcomes, including academic and perceived performance [8, 9, 32–35]. Although the use of learning styles has been contradictory, it is interesting to note that beliefs in the value of using learning styles are still popular among higher education faculty [10–12]. Therefore, many teachers have also endorsed the use of learning styles in our university and attempted to improve the quality of ICM classes via the use of learning styles. In the context of the ICM, our results first indicated that learning styles had no effect on students’ satisfaction and could not predict students’ satisfaction with the traditional or ICM approach, which was consistent with experimental studies disproving the learning styles hypothesis. Thus, learning styles may not be a potential contributing factor for optimizing the application of the ICM to improve our educational services.

In addition to overall satisfaction, our study found that learning styles did not affect the students’ satisfaction with each of the four components of the ICM, which further supported findings that the correlation between learning styles and students’ satisfaction with different instruction approaches is small. All of the participants in the ICM class were more satisfied with the online course and class-meeting components than the student group discussion and presentation components. The possible reason for the lower satisfaction with components 2 and 3 may be the fear of feeling lost or unprepared, as captured by the following items in the questionnaire: “I fear public speaking because I might give a wrong answer” and “I want more guidance.” As Cristina R. noted, the added workload (e.g., the concept of “teaching ourselves” that led students to believe they had “extra” work) and the fear of unsettled classrooms (e.g., that group discussions and problem-solving activities such as presentations may create a chaotic classroom environment) are reasonable fears resulting from long-term adaptation to learning under traditional instruction [20]. Our study showed that students also had fears about the ICM approach, which have been reported in the literature [36–38]. These fears can be overcome in the long-term by increasing teaching commitment from instructors in ICM classes. Therefore, instead of focusing on the use of learning styles, it may be more productive for teachers and students to consider how to help students overcome feelings of fear regarding ICM classes.

The mean satisfaction scores for the ICM class in our study were significantly higher than those for the traditional class, regardless of the students’ learning styles, which is similar to many reports that have shown higher satisfaction ratings for ICM classes among medical students [39–43]. These results possibly arise from an agreement between the demands of medical studies and the characteristics of the ICM. It has been well accepted that the ICM features active learning, critical thinking, communication ability and so forth, which are aimed to enhance learning and professional development in medical students [2, 20, 42]. The results show that the participants in the ICM class gave higher scores than the participants in the traditional class for the statement “The course format is helping me to prepare for my future professional life better”; the highest satisfaction score (3.9) was given by participants in the ICM approach in response to this statement. In the written responses, one of the students in the ICM class wrote, “I made some improvements in communication skills and critical thinking through this course,” which are necessary abilities in a successful physician’s career [44]. The results of the present study also confirmed that the ICM approach might be more likely to improve these skills than the traditional approach. However, the participants in the traditional class gave higher scores than the participants in the ICM class for the statement “I spend less time on learning in this course”; the highest satisfaction score (3.5) given by participants in the traditional class was given in response to this statement. The only negative result showed that the participants in the ICM class were less satisfied with the time spent on the pre-course component (MOOCs) than participants in the traditional approach. Although research on ICM courses has become more prevalent [45], one major challenge raised regarding MOOCs is low-course completion rates and high dropout rates [46, 47]; one of the causes for these problems is a lack of time, which was noted by Belanger & Thornton [48]. Our results also indicated that students in the ICM class probably spent much more time completing the course correctly than those in the traditional approach. Therefore, instead of focusing on the use of learning styles, further research on how to address this issue must focus on how to design more efficient online courses, determine appropriate levels of student learning materials, and provide more online instructional interaction.

A limitation of this study is the small number of questions in the satisfaction questionnaire. The 10 items were developed based on the CEQ used in Australia [22] and referenced a previously published empirical research paper [16], which could be feasible to evaluate the students’ satisfaction with courses; however, adding more new items that reflect the impact of the ICM on active learning, critical thinking, and communication ability may provide more comprehensive results. However, developing a new questionnaire with high reliability would require much work in the future. Another limitation of our study is that there was a three-year gap between the two courses that were compared. Although the same teaching syllabus, textbook and learning materials, such as the problem sets, have been used for the past five years, a better comparison method could be self-reported, pre- and post-control observations or a group comparison of students of the same grade. To eliminate the effects of two different courses on the evaluation of teaching approaches, we used ANCOVA (analysis of covariance) before the comparison to confirm that the different two courses, that is, the covariates, would not affect our results for the dependent variable (data not shown). However, there must be bias due to the time lag. Therefore, longitudinal studies with the same groups could provide more convincing results.

Another limitation of this study was the lack of an effect of ICM vs. traditional teaching on academic achievement. We analyzed the relationship between learning style and learning outcome, but there was no statistically significant difference between the exam scores of the four learning style groups, and in the logistic regression analysis, none of the four learning styles were predictors of student success in either the traditional or ICM group (data not shown).

## Conclusions

Our results showed that the majority of the participants were convergers and assimilators. Learning style did not influence participants’ satisfaction with different instruction approaches and components of the ICM; thus, learning style may not predict satisfaction with traditional and ICM approaches. Participants were more satisfied with the ICM than the traditional approach, regardless of which learning styles they favoured. Our findings lead us to believe that learning style may not be a potential contributing factor for optimizing the application of the ICM to improve our educational services. The results showed lower satisfaction with the time required for learning in the ICM and the student group discussion and presentation components, which revealed that some weaknesses exist in the ICM currently employed in our university. Instead of focusing on learning styles, further research should explore how to design more efficient online courses, determine appropriate levels of learning materials, provide more online instructional interaction and help students overcome feelings of fear.

## Additional files

Additional file 1: **Figure S1.** Distribution of participants according to Kolb’s learning style model. (PDF 8 kb)

Additional file 2: **Table S1**. Typical learning activities in the general physiology part of the ICM class. (PDF 105 kb)

Additional file 3: **Table S2**. Tests of between-subjects effects (n = 121). # P < 0.05, analysis by general linear model-based univariate ANOVA. (PDF 57 kb)

Additional file 4: **Table S3**. Binary logistic regression of the relationship between satisfaction and learning styles in the traditional and ICM approaches. Bivariable logistic regression analysis of learning styles and teaching approach satisfaction among second-year dental students at Cheeloo College of Medicine, Shandong University, China 2018 (n = 121); *CI* = confidence interval. (PDF 96 kb)

Additional file 5: **Table S4**. Tests of between-subjects effects (n = 63). Analysis by general linear model-based multivariate ANOVA. (PDF 87 kb)

## Data Availability

The dataset used during the study is available from the corresponding author on reasonable request.

## Abbreviations

AC: Abstract conceptualization
AE: Active experimentation
CE: Concrete experience
F2F: Face to face
ICM: Inverted classroom model
LSI: Kolb’s Learning Style Inventory
MOOC: Massive open online course
RO: Reflective observation

## References

[1] Newble DI, Gordon MI (1985). The learning style of medical students. Med Educ.

[2] Tolks D, Schafer C, Raupach T, Kruse L, Sarikas A, Gerhardt-Szep S, Kllauer G, Lemos M, Fischer MR, Eichner B *et al*: An Introduction to the Inverted/Flipped Classroom Model in Education and Advanced Training in Medicine and in the Healthcare Professions. *GMS J Med Educ* 2016, 33(3):Doc46. 10.3205/zma001045 PMC4894356 27275511

[3] Langer VL, Knut & Schimanke, Florian.: improvement of self-directed learning by using the inverted classroom model (ICM) for a basic module in business computer sciences. 10.13140/2.1.3620.5768. . In: *The 3rd German ICM-Conference.* Marburg,Germany; 2014.

[4] Mehta NB, Hull AL, Young JB, Stoller JK (2013). Just imagine: new paradigms for medical education. Acad Med.

[5] McLaughlin JE, Roth MT, Glatt DM, Gharkholonarehe N, Davidson CA, Griffin LM, Esserman DA, Mumper RJ (2014). The flipped classroom: a course redesign to foster learning and engagement in a health professions school. Acad Med.

[6] Prober CG, Khan S (2013). Medical education reimagined: a call to action. Acad Med.

[7] Pashler H, McDaniel M, Rohrer D, Bjork R (2008). Learning styles: concepts and evidence. Psychol Sci Public Interest.

[8] Rohrer D, Pashler H (2012). Learning styles: where's the evidence?. Med Educ.

[9] Newton PM, Miah M (2017). Evidence-based higher education - is the learning styles 'Myth' important?. Front Psychol.

[10] Simmonds A: How Neuroscience Is Affecting Education: Report of Teacher and Parent Surveys. In*.* https://wellcome.ac.uk/sites/default/files/ wtp055240.pdf 2014.

[11] Dandy KBK. Student and faculty beliefs about learning in higher education: implications for teaching. Int J Teach Learn High Educ. 2014;26.

[12] Newton PM (2015). The learning styles myth is thriving in higher education. Front Psychol.

[13] Coffield F, Moseley, D., Hall, E., & Ecclestone, K.: Learning styles and pedagogy in post-16 learning. A systematic and critical review. In*.* London: Learning and Skills Research Centre.; 2004.

[14] Kolb DA (1976). The learning style inventory: technical manual. , Boston, MA. McBer & co.

[15] Dekker SLN, Howard-Jones P, Jolles J. Neuromyths in education: prevalence and predictors of misconceptions among teachers. Front Psychol. 2012;3. 10.3389/fpsyg.2012.00429 PMC3475349 23087664

[16] Gurpinar E, Alimoglu MK, Mamakli S, Aktekin M (2010). Can learning style predict student satisfaction with different instruction methods and academic achievement in medical education?. Adv Physiol Educ.

[17] Pungente MD, Wasan KM, Moffett C (2003). Using learning styles to evaluate first-year pharmacy Students' preferences toward different activities associated with the problem-based learning approach. Am J Pharm Educ.

[18] Day LJ (2018). A gross anatomy flipped classroom effects performance, retention, and higher-level thinking in lower performing students. Anat Sci Educ.

[19] Morgan H, McLean K, Chapman C, Fitzgerald J, Yousuf A, Hammoud M (2015). The flipped classroom for medical students. Clin Teach.

[20] Rotellar C, Cain J (2016). Research, perspectives, and recommendations on implementing the flipped classroom. Am J Pharm Educ.

[21] Xiao N, Thor ZM, Baek J, Kim G (2018). Flipped classroom narrows the performance gap between low- and high-performing dental students in physiology. Adv Physiol Educ.

[22] Course Experience Questionnaire [https://planning.curtin.edu.au/local/docs/CEQ_questionnaire2010.pdf].

[23] Engleberg NC, Schwenk T, Gruppen LD (2001). Learning styles and perceptions of the value of various learning modalities before and after a 2nd-year course in microbiology and infectious diseases. Teach Learn Med.

[24] Schroder H, Henke A, Stieger L, Beckers S, Biermann H, Rossaint R, Sopka S (2017). Influence of learning styles on the practical performance after the four-step basic life support training approach - an observational cohort study. PLoS One.

[25] Kolb DA (1981). Learning styles and disciplinary differences.

[26] Carey K, Cambiano, R. & De Vore, J., : Student to faculty satisfaction at a Midwestern university in the USA. 2002: 93–97.

[27] Mukhtar U, Anwar S, Ahmed U, Baloch MA (2015). Factors effecting the service quality of public and private sector universities comparatively: an empirical investigation.

[28] Alghasham AA (2012). Effect of students' learning styles on classroom performance in problem-based learning. Med Teach.

[29] E P (2010). Adaptation provisioning with respect to learning styles in a web-based educational system: an experimental study. J Comput Assist Learn.

[30] Y H (2012). The effect of teaching methods and learning style on learning program design in web-based education systems. J Educ Comput Res.

[31] Smits PB, Verbeek JH, Nauta MC, Ten Cate TJ, Metz JC, van Dijk FJ (2004). Factors predictive of successful learning in postgraduate medical education. Med Educ.

[32] JA ASaH (2010). Learning styles in the classroom: educational benefit or planning exercise?. Psychology Teaching Review.

[33] Norman G (2009). When will learning style go out of style?. Adv Health Sci Educ Theory Pract.

[34] RE M (2011). Does styles research have useful implications for educational practice?. Learn Individ Differ.

[35] Kappe F.R., Boekholt L., den Rooyen C., Van der Flier H. (2009). A predictive validity study of the Learning Style Questionnaire (LSQ) using multiple, specific learning criteria. Learning and Individual Differences.

[36] Lage MJPG, Treglia M (2003). Inverting the classroom: a gateway to creating an inclusive learning environment. J Econ Educ.

[37] Sharma NLC, Doherty I, Harbutt D (2015). How we flipped the medical classroom. Med Teach.

[38] Smith J (2013). Student attitudes toward flipping the general chemistry classroom. Chem Educ Res Pract.

[39] Lewis CE, Chen DC, Relan A (2018). Implementation of a flipped classroom approach to promote active learning in the third-year surgery clerkship. Am J Surg.

[40] Wittich CM, Agrawal A, Wang AT, Halvorsen AJ, Mandrekar JN, Chaudhry S, Dupras DM, Oxentenko AS, Beckman TJ (2018). Flipped classrooms in graduate medical education: A National Survey of residency program directors. Acad Med.

[41] Belfi LM, Bartolotta RJ, Giambrone AE, Davi C, Min RJ: "flipping" the introductory clerkship in radiology: impact on medical student performance and perceptions *Acad Radiol* 2015, 22(6):794–801. 10.1016/j.acra.2014.11.003 25592027

[42] Peterson Daniel J. (2015). The Flipped Classroom Improves Student Achievement and Course Satisfaction in a Statistics Course. Teaching of Psychology.

[43] Burnham KD, Mascenik J (2018). Comparison of student performance and perceptions of a traditional lecture course versus an inverted classroom format for clinical microbiology. J Chiropr Educ.

[44] Berwick DM, Finkelstein JA (2010). Preparing medical students for the continual improvement of health and health care: Abraham Flexner and the new "public interest". Acad Med.

[45] Chen F, Lui AM, Martinelli SM (2017). A systematic review of the effectiveness of flipped classrooms in medical education. Med Educ.

[46] A-ÖE BA, Zawacki-Richter O. Trends and patterns in massive open online courses: review and content analysis of research on MOOCs (2008–2015). Int Rev Res Op Dis Lear. 2017;18(5):119–47.

[47] Khalil HEM (2014). MOOCs completion rates and possible methods to improve retention - a literature review. AACE.

[48] Belanger YT (2014). J.: bioelectricity: A quantitative approach, Duke University’s first MOOC, Duke Center for instructional technology. *In Proceedings of World Conference on Educational Multimedia, Hypermedia and Telecommunications* Chesapeake, VA.

