# Supplementary figures and images for: The relation of dental students’ learning styles to their satisfaction with traditional and inverted classroom models
Source: BMC Med Educ. 2019 Aug 22;19:315. doi: 10.1186/s12909-019-1749-x (PMC6704638; doi:10.1186/s12909-019-1749-x)

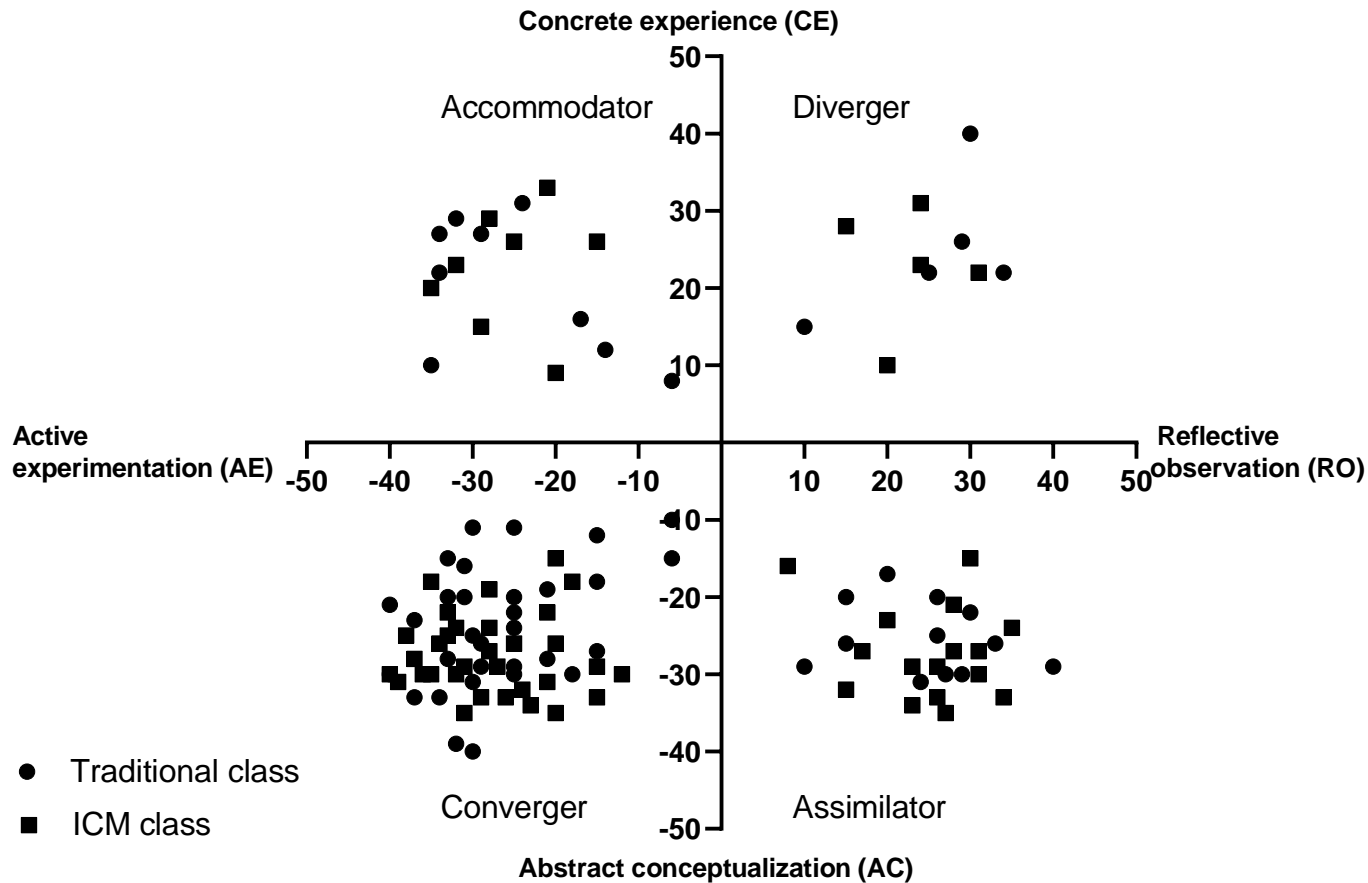

Supplement: Supplementary file 1 — Figure S1. Distribution of participants according to Kolb’s learning style model. (PDF 8 kb) [file 12909_2019_1749_MOESM1_ESM.pdf]
