# Supplementary material for: The relation of dental students’ learning styles to their satisfaction with traditional and inverted classroom models
Source: BMC Med Educ. 2019 Aug 22;19:315. doi: 10.1186/s12909-019-1749-x (PMC6704638; doi:10.1186/s12909-019-1749-x)
Supplement: Supplementary file 2 — Table S1. Typical learning activities in the general physiology part of the ICM class. (PDF 105 kb) [file 12909_2019_1749_MOESM2_ESM.pdf]

Thematic block	Topic	Online self-study	Online quiz	Classroom meeting
1st BLOCK<br>CELL	Cell physiology	PPT and other<br>Reading materials		A Lecture on cells, homeostasis, regulation, feedback controls etc.
Sub-block1	Transport of substances through cell membranes	Micro-lesson videos:<br>1. Cell membrane<br>2. Passive transport<br>3. Active transport<br>4. Cytosis	MCQs mainly based on first lecture topics and block1 self-study micro-lesson videos.	Group discussion/mind map show/ interaction with instructors
