# Supplementary material for: The relation of dental students’ learning styles to their satisfaction with traditional and inverted classroom models
Source: BMC Med Educ. 2019 Aug 22;19:315. doi: 10.1186/s12909-019-1749-x (PMC6704638; doi:10.1186/s12909-019-1749-x)
Supplement: Supplementary file 3 — Table S2. Tests of between-subjects effects (n = 121). # P < 0.05, analysis by general linear model-based univariate ANOVA. (PDF 57 kb) [file 12909_2019_1749_MOESM3_ESM.pdf]

Tests of Between-Subjects Effects

Dependent Variable: Satisfaction

Source	Type III Sum of Squares	df	Mean Square	F	Sig.
Corrected Model	11.534[a]	7	1.648	5.399	.000
Intercept	788.570	1	788.570	2583.837	.000
Learning Style	.602	3	.201	.657	.580
Teaching Approach#	9.455	1	9.455	30.981	.000
Learning Style * Teaching Approach	2.117	3	.706	2.312	.080
Error	34.487	113	.305		
Total	1419.060	121			
Corrected Total	46.021	120			

a. R Squared = .251 (Adjusted R Squared = .204)
