# Supplementary material for: The relation of dental students’ learning styles to their satisfaction with traditional and inverted classroom models
Source: BMC Med Educ. 2019 Aug 22;19:315. doi: 10.1186/s12909-019-1749-x (PMC6704638; doi:10.1186/s12909-019-1749-x)
Supplement: Supplementary file 4 — Table S3. Binary logistic regression of the relationship between satisfaction and learning styles in the traditional and ICM approaches. Bivariable logistic regression analysis of learning styles and teaching approach satisfaction among second-year dental students at Cheeloo College of Medicine, Shandong University, China 2018 (n = 121); CI = confidence interval. (PDF 96 kb) [file 12909_2019_1749_MOESM4_ESM.pdf]

Variables	Traditional approach		ICM approach	
	Odds Ratio (95% CI)	*P* Value	Odds Ratio (95% CI)	*P* Value
**Converger**	1	0.1	1	0.9
**Diverger**	0.2 (0.02, 1.8)	0.1	1.9 (0.2, 19.2)	0.6
**Accommodator**	0.3 (0.05, 1.2)	0.1	0.8 (0.2, 4)	0.8
**Assimilator**	1.5 (0.3, 6.7)	0.6	0.8 (0.2, 2.8)	0.7
