# Supplementary material for: The relation of dental students’ learning styles to their satisfaction with traditional and inverted classroom models
Source: BMC Med Educ. 2019 Aug 22;19:315. doi: 10.1186/s12909-019-1749-x (PMC6704638; doi:10.1186/s12909-019-1749-x)
Supplement: Supplementary file 5 — Table S4. Tests of between-subjects effects (n = 63). Analysis by general linear model-based multivariate ANOVA. (PDF 87 kb) [file 12909_2019_1749_MOESM5_ESM.pdf]

**Tests of Between-Subjects Effects**

| Source | Dependent Variable | Type III Sum of Squares | df | Mean Square | F | Sig. |
|---|---|---|---|---|---|---|
| Corrected Model | Statement 1 | 1.457[a] | 3 | .486 | 1.414 | .248 |
| | Statement 2 | 3.241[b] | 3 | 1.080 | 1.433 | .242 |
| | Statement 3 | 6.043[c] | 3 | 2.014 | 2.606 | .060 |
| | Statement 4 | 3.737[d] | 3 | 1.246 | 2.449 | .072 |
| Intercept | Statement 1 | 729.023 | 1 | 729.023 | 2123.296 | .000 |
| | Statement 2 | 417.977 | 1 | 417.977 | 554.510 | .000 |
| | Statement 3 | 363.360 | 1 | 363.360 | 470.053 | .000 |
| | Statement 4 | 574.840 | 1 | 574.840 | 1130.187 | .000 |
| Learning Style | Statement 1 | 1.457 | 3 | .486 | 1.414 | .248 |
| | Statement 2 | 3.241 | 3 | 1.080 | 1.433 | .242 |
| | Statement 3 | 6.043 | 3 | 2.014 | 2.606 | .060 |
| | Statement 4 | 3.737 | 3 | 1.246 | 2.449 | .072 |
| Error | Statement 1 | 20.257 | 59 | .343 | | |
| | Statement 2 | 44.473 | 59 | .754 | | |
| | Statement 3 | 45.608 | 59 | .773 | | |
| | Statement 4 | 30.009 | 59 | .509 | | |
| Total | Statement 1 | 1284.000 | 63 | | | |
| | Statement 2 | 809.000 | 63 | | | |
| | Statement 3 | 745.000 | 63 | | | |
| | Statement 4 | 1074.000 | 63 | | | |
| Corrected Total | Statement 1 | 21.714 | 62 | | | |
| | Statement 2 | 47.714 | 62 | | | |
| | Statement 3 | 51.651 | 62 | | | |
| | Statement 4 | 33.746 | 62 | | | |

a. R Squared = .067 (Adjusted R Squared = .020)

b. R Squared = .068 (Adjusted R Squared = .021)

c. R Squared = .117 (Adjusted R Squared = .072)

d. R Squared = .111 (Adjusted R Squared = .066)
